# Cytological Observations of the Large Symbiotic Foraminifer *Amphisorus kudakajimensis* Using Calcein Acetoxymethyl Ester

**DOI:** 10.1371/journal.pone.0165844

**Published:** 2016-11-03

**Authors:** Yoshikazu Ohno, Kazuhiko Fujita, Takashi Toyofuku, Takashi Nakamaura

**Affiliations:** 1 Marine and Environmental Sciences Course, Graduate School of Engineering and Science, University of the Ryukyus, Senbaru 1, Nishihara, Okinawa 903-0213, Japan; 2 Department of Physics and Earth Sciences, Faculty of Science and Tropical Biosphere Research Center, University of the Ryukyus, Senbaru 1, Nishihara, Okinawa 903-0213, Japan; 3 Department of Marine Biodiversity Research (B-DIVE), Japan Agency for Marine-Earth Science and Technology (JAMSTEC), 2-15 Natsushima-cho, Yokosuka 237-0061, Japan; 4 Department of Chemistry, Biology and Marine Science, Faculty of Science and Tropical Biosphere Research Center, University of the Ryukyus, Senbaru 1, Nishihara, Okinawa 903-0213, Japan; 5 Japan Science and Technology Agency (JST)/ Japan International Cooperation Agency (JICA) SATREPS, Tokyo Headquarters, 8th Floor, K's Gobancho 7, Gobancho, Chiyoda-ku, Tokyo 102-0076, Japan; Bernhard Nocht Institute for Tropical Medicine, GERMANY

## Abstract

Large benthic foraminifera are unicellular calcifying reef organisms that can form symbiotic relationships with a range of different microalgae. However, the cellular functions, such as symbiosis and calcification, and other aspects of cellular physiology in large benthic foraminifera are not fully understood. *Amphisorus kudakajimensis* was used as a model to determine the detailed cellular characteristics of large benthic foraminifera. We used calcein acetoxymethyl ester (calcein AM) as a fluorescent indicator for live confocal imaging. We demonstrated that calcein AM is a useful fluorescent indicator to stain the fine network of reticulopodia and the cytoplasm in living *A*. *kudakajimensis*. We showed that at least two types of reticulopodia exist in *A*. *kudakajimensis*: the straight bundle of reticulopodia that spreads from the aperture and the fine reticulopodia along the surface of the aperture and chamber walls. The cytoplasm in outer chambers was highly branched and contained a few dinoflagellates. In contrast, the inner chamberlets contained condensed cytoplasm and many dinoflagellates, suggesting that the cytoplasm of *A*. *kudakajimensis* performs different functions based on its location within the large test. Our confocal detailed image analysis provides real-time cellular morphology and cell physiology of living foraminifera.

## Introduction

Foraminifera are single-celled protists, and modern taxonomies rank the group as a phylum or subphylum [1]. Foraminifera typically produce a shell, known as a test, which can have one or multiple chambers; indeed, some tests can become quite elaborate in structure. The shells are commonly made of calcium carbonate (CaCO_3_) or agglutinated sediment particles [2]. Foraminifera first appeared in the Cambrian and have since radiated into tens of thousands of species during Phanerozoic, including approximately 4000 extant species living today [3]. In addition, they are a major group in the microfossil record, and derived micropaleontological data have been extensively utilized for biostratigraphic, paleoenvironmental, and paleoceanographic interpretations [4–5].

The cytological feature of foraminifera is characterized by their threadlike, granule-containing pseudopodia (i.e., granular reticulopodia) that stream bidirectionally. The spreading of reticulopodia from the main part of cytoplasm comprises the main mechanism with which foraminifera interact with their environment [6]. The main functions of reticulopodia include food capture [7], motility and attachment [8–9], test construction [6, 10–11], and, possibly, some aspects of reproduction [12–13]. These cellular functions of reticulopodia have been revealed mainly by using scanning and transmission electron microscopy. The electron microscopy has also revealed motile microtubule networks [8, 14], organelle distribution [15–17], and cytoplasmic morphology [18–19]. However, the high vacuum conditions required for this technique prohibits the study of living specimens in vivo. Because foraminifera have translucent reticulopodia and opaque cytoplasm, effective observation can be problematic, hindering further knowledge growth regarding deeper cellular function and cytoplasmic behavior.

Recently, fluorescent live-imaging techniques have been employed in cytological studies of foraminifera. This microscopic technique has the great advantage of high-resolution observations within living foraminifera. The pH gradient within foraminifera was measured using pH-sensitive fluorescent dyes [20–22]; in addition, dyes were used to study calcium accumulation in order to better understand the calcification processes [23–24]. The elevation of pH in compartmentalized seawater-containing vacuoles [20–22] and calcium ion accumulation were observed during chamber formation in living foraminifera [23–24].

In this study, we performed a simple staining method to visualize the cytoplasm and reticulopodia within live *Amphisorus kudakajimensis* (Gudmundsson, 1994), which is a large benthic foraminifera (LBF) with a porcelaneous shell [25]. For this purpose, we utilized calcein acetoxymethyl ester (calcein AM). The chemical property of non-fluorescnet cacein AM is different to fluorescent calcein. Fluoresent dye calcein is a widely used fluorescent indicator that stains hard tissue in marine organisms, including foraminifera [26–28]. On the other hand, calcein AM permeates the plasma membrane and, in living cells, is hydrolyzed to calcein, which is a green, highly fluorescent, negatively charged, and stable molecule with low toxicity [29]. Fluorescent calcein is insensitive to pH and intracellular ion concentrations, and it does not undergo spectral changes after accumulation in intracellular compartments or when bound to cellular components [29]. Notably, dead cells lack an active esterase enzyme, and thus only live cells are labeled by calcein. Based on these chemical properties, calcein AM has a broad range of applications in physiological studies, such as viability assays [30], cytotoxicity assays [31], cell volume studies [32], and the study of chemotaxis [33].

## Materials and Methods

### Target species

*Amphisorus kudakajimensis* [25] was used in this study. This species belongs to the genus *Amphisorus*, which is one of algal symbiont-bearing LBF that thrive in modern coral reef environments. *A*. *kudakajimensis* hosts dinoflagellate symbionts. The discoid porcelain-like shell with thickened peripheries is characterized by numerous median apertures between the marginal apertural rows [34]. The horizontal arrangement of numerous cyclic chambers with relatively transparent chamber walls makes this species ideal for observing cytoplasm with confocal imaging.

### Ethics statement

Specific permission was not required to collect and use living *A*. *kudakajimensis* in our experiments conducted in the Okinawa prefecture in Japan. *A*. *kudakajimensis* is one of the most common symbiont-bearing LBFs on the reef flats of Okinawa [34], and it is not an endangered or protected species.

### Sampling and culture methods

*A*. *kudakajimensis* specimens were collected during low tide from nearshore reef flats at Bise (26°70'N, 127°88'E), Motobu-cho, Okinawa, Japan between June 2014 and July 2015. Individual specimens approximately 1.5–4.0 mm in diameter were removed slowly from the substratum using a brush. Next, the specimens were gently washed, placed in plastic vessels (10–15 specimens in 0.5 L of seawater), and brought back to the laboratory. After collection, sets of 10–15 specimens were maintained in glass vessels containing 0.5 L of filtered seawater (FSW; 1.0 μm pore size, pH = 8.1, salinity = ca. 35, collected from the Sesoko Station close to the sampling site) at room temperature (25.5 ± 0.5°C) under a 12-h light/dark cycle (approximately 40 μmol photons/m^2^ s). The illumination level was chosen based on previous experiments [35]. The specimens were not fed because the digestive remnants can create artifacts during microscopic observation. The specimens were kept alive for more than 1 month until the experiments were completed, and the FSW was changed weekly.

### Confocal imaging

We used two confocal imaging systems. The spinning-disk confocal imaging system was comprised of a Nikon Eclipse Ti-U inverted epifluorescence microscope (Tokyo, Japan) with a hand-made reflection light, a Yokogawa laser-scanning unit (CSU-X1; Tokyo, Japan), and a Hamamatsu Photonics ImagEM C9100-13 electron-multiplying charge-couple device camera (Hamamatsu, Japan). This equipment was operated by a Hamamatsu Photonics AQUACOSMOS/RATIO system (Hamamatsu, Japan). The second confocal imaging system consisted of a Nikon A1+ confocal microscope (Tokyo, Japan) equipped with a high-resolution galvano scanner and was operated using Nikon NIS Elements software (Tokyo, Japan). Detailed optical sections were obtained approximately 50–200 μm from the surface of the test. These sections were reconstructed as z-stack images with NIS Elements software (Nikon).

### Fluorescent dye (calcein AM)

Calcein AM, a nonfluorescent and cell-permeable compound, is converted into the highly green fluorescent calcein when hydrolyzed by intracellular esterases in a living cell. We assessed calcein accumulation over time in *A*. *kudakajimensis* to determine an appropriate incubation period and dye concentration (S1 Appendix). According to the results, a stock solution of calcein AM (1 mM in dimethyl sulfoxide; Dojindo Molecular Technologies, Kumamoto, Japan) was diluted with FSW to a final concentration of 10 μM. Filter wavelengths of 488 nm for excitation and 500–550 nm for emission were used to detect hydrolyzed calcein in the cytoplasm.

### Cytoplasm observations in living *A*. *kudakajimensis*

To observe morphological differences in the cytoplasm, we classified the chambers of *A*. *kudakajimensis* into four regions according to a previous study: outer chambers, intermediate chambers, inner chambers, and the embryonic apparatus [36]. The specimens were incubated in 10 μM calcein AM for 30 min at room temperature. Each specimen was placed on a glass-based dish (No. 1S, thickness = 0.15–0.18 mm; Iwaki Glass, Tokyo, Japan). Experiments were conducted within 1 h after recovery of each specimen to prevent the contraction of reticulopodia due to stimulation by rapid environmental changes. We simultaneously obtained images of the calcein-labeled cytoplasm, the distribution of symbiotic algae, and the corresponding bright-field image. Autofluorescence from symbiotic algae was detected with an excitation wavelength of 561 nm and an emission wavelength of 580–640 nm.

To confirm that dead cells were not labeled by calcein AM, we used a 10% formaldehyde FSW solution for cell fixation. After incubation for 24 h, the fixation solution was washed out completely. Next, the foraminifera were incubated with a calcein AM as described above. To visualize the aperture of the specimen, observations were made with the organism in an upright position. All of the images were captured while the specimens were under water.

## Results and Discussion

### Live/dead staining pattern of calcein AM

**Fig 1:**
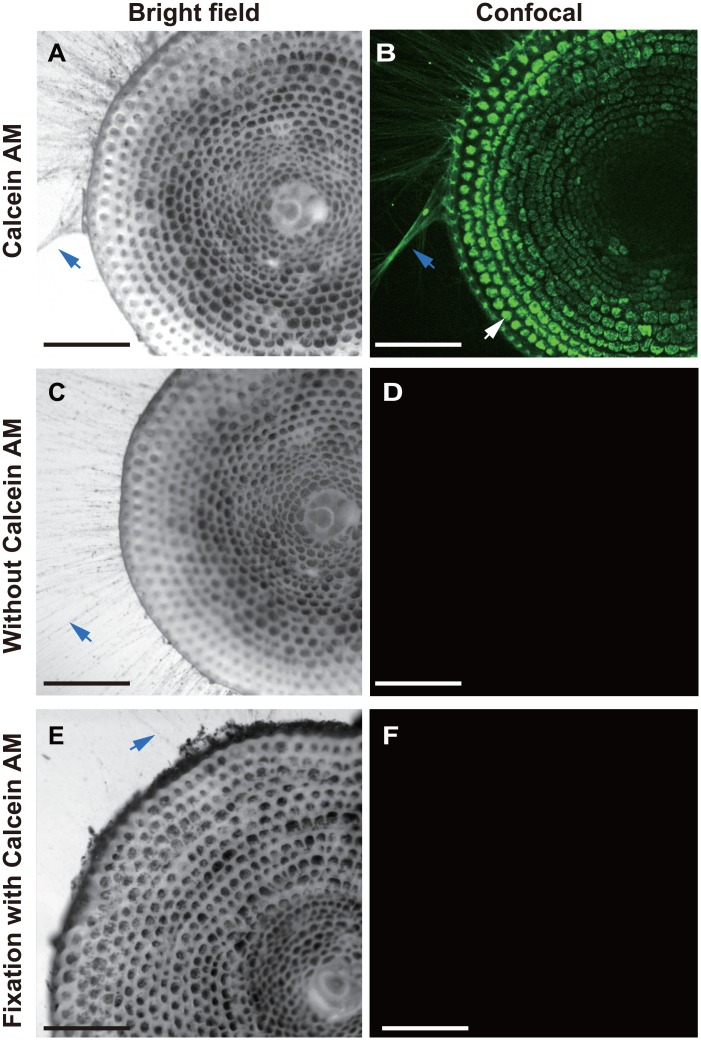
Cytoplasm and reticulopodia of *Amphisorus kudakajimensis* observed using bright-field and confocal imaging with calcein AM. An example of the discrimination between live and dead *A*. *kudakajimensis* with calcein AM staining. **(A, B)** Living specimens stained with calcein AM (10 μM). **(C, D)** Living specimens without calcein AM. **(E, F)** Dead *A*. *kudakajimensis* stained with calcein AM (10 μM). **(A, C, E)** Bright-field images. **(B, D, F)** Confocal images. The blue arrows indicate the spreading reticulopodia, and white arrow indicates the cytoplasm in chamberlets. All scale bars = 200 μm.

Bright-field images of *A*. *kudakajimensis* showed the spreading of reticulopodia outside of the test and the localization of numerous dinoflagellates to inner parts of the test, which were dark in appearance. However, we could not obtain a clear image of the cytoplasm in each of the chamberlets in the outer chambers (Fig 1A). Using calcein AM, the intrashell cytoplasm and reticulopodia bundles in live *A*. *kudakajimensis* were clearly visible (green fluorescence, Fig 1B). The calcein signal from the cytoplasm in the approximately last four chambers from the periphery, apertures, and reticulopodia bundles was stronger than the signal from the cytoplasm in the inner chambers (Fig 1B). This staining pattern was the result of a high density of dinoflagellates in the cytoplasm of the inner chambers that reduced the calcein fluorescence. Green auto-fluorescence was absent in *A*. *kudakajimensis* that were not treated with calcein AM (Fig 1D).

The radial spreading of reticulopodia in bright-field images was similar in specimens treated with (Fig 1A) or without (Fig 1C) calcein AM. Thus, calcein AM, up to a concentration of 10 μM, can be used to stain the cytoplasm in *A*. *kudakajimensis* without serious adverse effects (also see S1 Appendix). All of the specimens survived for more than 1 month after incubation with calcein AM (n = 6, where n denotes the number of specimens observed).

To show that calcein AM stains only live *A*. *kudakajimensis*, we performed calcein AM staining with dead specimens after fixation with 10% formaldehyde. Notably, the reticulopodia and dinoflagellates in the cytoplasm of the chambers remained (Fig 1E). The fixed specimens lost their intrashell cytoplasmic streaming, as well as their reticulopodia activity (n = 6) (data not shown). Although all of the live specimens were stained with calcein AM (Fig 1B) (n = 6), no fluorescent calcein signal was detected in dead specimens (Fig 1F). Our experimental data suggest that calcein AM can be used to discriminate between the cytoplasm of living and dead foraminifera after a short incubation (see S1 Appendix).

A previous study used a different fluorescent dye, CellTracker Green CMFDA, to determine its capability as a detection method for living foraminifera [37–39]. Once this membrane-permeant probe enters a cell, esterases hydrolyze the nonfluorescent CMFDA to fluorescent 5-chloromethylfluorescein, which then reacts with thiols in proteins and peptides to form aldehyde-fixable conjugates [40]. The dye is retained in the cytoplasm of living foraminifera, and it can be formalin fixed in the specimen with limited artifacts when compared with the conventional Rose Bengal staining method [39]. Calcein AM is comparable to CellTracker Green CMFDA for the observation of the cytoplasm over a period of a few hours. However, hydrolyzed calcein was not uniformly retained in the cytoplasm 24 h after incubation (see S1 Appendix), suggesting that calcein is gradually discharged from live *A*. *kudakajimensis* cells. Particularly, calcein AM with its short incubation time may be useful as a viability assay of living foraminifera in culture experiments that require relatively quick incubations with dye.

### Reticulopodia

**Fig 2:**
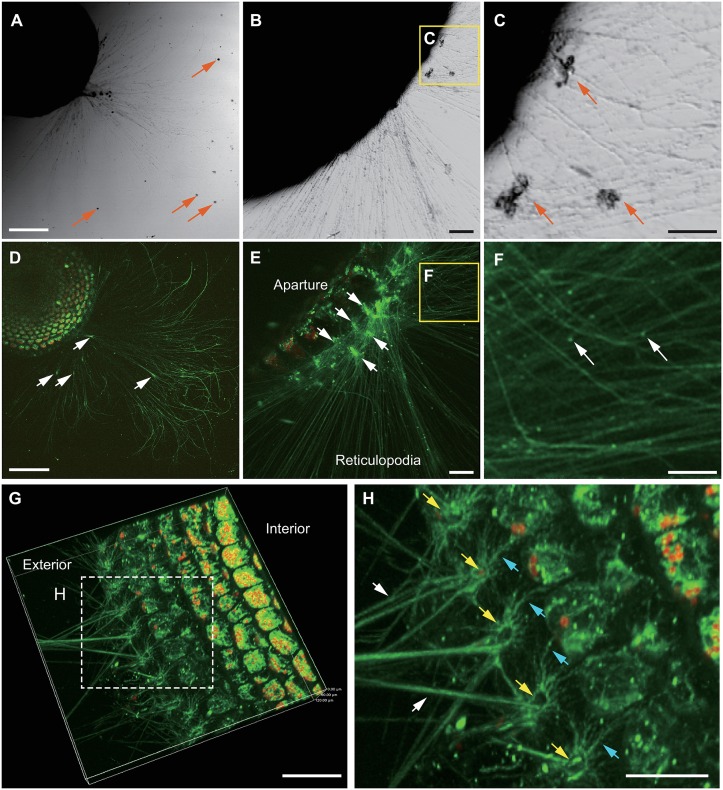
Bright-field and confocal images of *A*. *kudakajimensis* with spreading reticulopodia. **(A, B, C)** Transmitted bright-field images, **(D, E, F, G, H)** Confocal images. **(A)** Low-magnification images of *A*. *kudakajimensis* with spreading reticulopodia. Orange arrows point to excrement. Scale bar = 400 μm. **(B)** Image of the same specimen from (A) in a vertical position. The base of the specimen (aperture) was shaded under trans-illumination. Scale bar = 100 μm. **(C)** High-magnification image of the box shown in (B). Orange arrows indicate excrement. Scale bar = 50 μm. **(D)** Confocal image corresponding to the bright-field image in (A). The reticulopodia and cytoplasm are shown in green, and the dinoflagellates are shown in red. White arrows indicate bright spots in the reticulopodia that are attached to the glass substrate. Time-lapse images show streaming reticulopodia (S1 Movie). Scale bar = 400 μm. **(E)** High-magnification image of the aperture area corresponding to the image in (B). Scale bar = 100 μm. **(F)** High-magnification image of the reticulopodia corresponding to the image in (C). Scale bar = 50 μm. **(G)** Three-dimensional image (depth = 120 μm) of reticulopodia and the cytoplasm at the outermost part of the test. Scale bar = 400 μm. 3D rotation is shown in S2 Movie. **(H)** High-magnification three-dimensional image of the box shown in (G). Thick reticulopodia (white arrows) and fine reticulopodia (blue arrows) emerged from the marginal apertures. The ring-shape reticulopodial network around the marginal apertures was indicated with yellow arrows. Scale bar = 200 μm.

Low magnification images obtained with transmitted illumination showed reticulopodia spreading from the *A*. *kudakajimensis* test. The reticulopodial network approximately 1–2 cm away from the test was obscure (Fig 2A); however, the confocal image clearly showed reticulopodial network emerging from the test and extending to the tip of reticulopodia (Fig 2D) and the cytoplasm inside the test. The movement of reticulopodia on the glass (e.g., streaming, branching, and fusing) was captured using time-lapse imaging (S1 Movie). Live confocal imaging also detected bright spots in the reticulopodia strands (Fig 2D; white arrows), suggesting that the condensed reticulopodia were attached to the glass (S1 Movie). This observational data was quite similar to a study on the distribution of actin fibrils in reticulopodia [9]. The bright spots in reticulopodia may play a role in adhesive strength during migration; however, we cannot confirm the exact function of these bright spots or the fine structure of reticulopodia with the current data (i.e., the distribution actin fibrils). Further studies are needed to compare the calcein AM staining pattern and the distribution of actin fibrils.

Higher magnification of transmitted illumination images show the granules in reticulopodia (Fig 2B and 2C), whereas the fine details of the reticulopodia had a smooth appearance in the confocal image (Fig 2E and 2F). The difference in visualization may be due to the chemical characteristics of calcein AM. Transmitted illumination contrasted the fine structure of reticulopodia, whereas hydrolyzed calcein can be uniformly distributed in the cytosol. The reticulopodia granules were composed of mitochondria, food, and excrement [6]. Based on the chemical properties of calcein AM, inorganic objects, such as sand and excrement, are not stained. Indeed, the particles that likely correspond to excrement (Fig 2A and 2C; orange arrows) were not observed with confocal imaging (Fig 2D and 2F). Distinct small granules were observed in confocal images (e.g., Fig 2F; white arrows). Their location was hardly detected in the bright-field image (Fig 2C). We assume that these bright granules reflected the live microorganism trapped by reticulopodia. Calcein AM is a well-known fluorescent dye for live bacteria staining, such as in oral biofilm [41].

It is difficult to observe reticulopodia located at the aperture with transmitted illumination (Fig 2A–2C) and reflection light microscopy (Fig 1A–1C), because this observation method does not detect the transparent cytoplasm at the test surface nor illuminate the partially shadowed region under the test. Inverted confocal imaging clearly revealed the reticulopodial distribution in these area (Fig 2E). The confocal image of the apertural face of *A*. *kudakajimensis* was obtained when the *A*. *kudakajimensis* specimen adhered to the glass (in an upright position); however, this region was shaded by transmitted light (Fig 2B). Inverted confocal microscopy revealed rows of multiple apertures (Fig 2E; white arrows), as well as the straight bundles of reticulopodia extending from the apertures. The red fluorescence from dinoflagellates was also shown in the outermost chamberlets.

**Fig 3:**
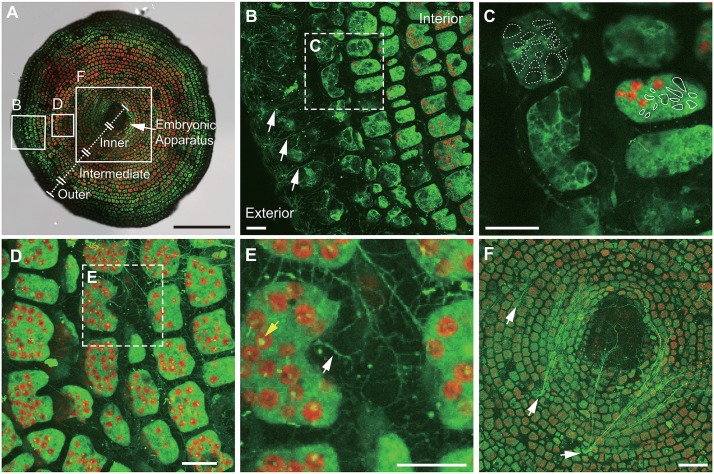
Confocal images of the intratest cytoplasm and reticulopodia covering the test surface of *A*. *kudakajimensis*. **(A)** Superimposed macro-confocal and bright-field images (Z-stack image at a depth of approximately 150 μm within the area of interest). The boxed areas in (A) correspond to the enlargement image in (B), (D) and (F). Four compartments were defined in *A*. *kudakajimensis*: outer chamber, shown in (B) and (C); intermediate and inner chambers, shown in (D) and (E); and inner chamber and embryonic apparatus, shown in (F). Scale bar = 1 mm. **(B)** The outer chambers consisted of approximately 6–8 rows from the periphery. The white arrows indicates fine and radically spreading reticulopodia extending from the aperture. Scale bar = 50 μm. **(C)** High-magnification image of the outer chambers from the dotted-line box shown in (B). The white encircled area indicates vacuoles. Cytosolic streaming is shown in S3 Movie. Scale bar = 50 μm. **(D)** High-magnification image of the intermediate to inner chambers from the boxed area shown in (A). Z-stack image of the intermediate chambers at a depth of approximately 50 μm. **(E)** High-magnification image of the boxed region shown in (D). The white arrow shows the fine reticulopodial network covering the test surface. The yellow arrow indicates the yellowish-green autofluorescence from dinoflagellates. Time-lapse imaging was performed in a single focal plane to visualize cytoplasmic streaming (S4 Movie). Scale bar = 50 μm. **(F)** Z-stack image of the inner chambers and embryonic apparatus at a depth of approximately 150 μm. Reticulopodia can be seen emerging at the base of the inner chambers (white arrows). Scale bar = 200 μm.

We also observed spreading reticulopodia bundles at the inner test that emerged form broken chamber walls (Fig 3F; white arrows), suggesting that any part of the cytoplasm in *A*. *kudakajimensis* has an ability to form reticulopodia, regardless of their position in the test. This cellular characteristic can be explained by the local control of pseudopodia organization derived from the secondary centers of networks [42–43]. The function of ectopic reticulopodia remains unknown; however, they may be used to increase adhesiveness and motility, even if the test wall was broken under natural conditions.

### Fine reticulopodia

To show the reticulopodial network from the aperture, we created a three-dimensional image using confocal microscopy (depth = 120 μm) (Fig 2G and S2 Movie). At high magnification, we observed thick and straight bundles of reticulopodia (Fig 2H; white arrows) and fine and radically spreading reticulopodia at the apertural periphery (Fig 2H; blue arrows). Fine reticulopodia were observed even when the thick bundle of reticulopodia was retracted into the test (Fig 3B; white arrows).

Highly branched, fine reticulopodia covering the test surface was also observed at the base of the intermediate chambers (Fig 3D and 3E; white arrow); however, this fine reticulopodia maintained a fixed position and showed cytoplasmic streaming (see S3 Movie, n = 3). Fine reticulopodia have been previously observed on the test surface of *Allogromia lalicollaris* and *Globorotalia truncatulinoides* [44–45]; however, the specific functions of fine reticulopodia at the apertures remain unknown because these studies observed fixed specimens under SEM. We speculate that these fine reticulopodia play a role in stabilizing adhesion for attachment to the surfaces of substrates. Additionally, fine reticulopodia may help to maintain the test surface (e.g., feeding on bacteria [46], protecting from harmful microorganisms, and restoration of broken chamber walls).

The fine reticulopodia formed a ring-shaped structure around the edge of the aperture (Fig 2H; blue arrows). The thick bundles of reticulopodia (Fig 2H; white arrows) did not close the apertures (Fig 2H; yellow arrow). These structural features are somewhat similar to the aperture of *Allogromia lalicollaris* (e.g., circumoral cytoplasm or peduncle) [44]. However, the cytoplasm (the basis of reticulopodia) of *A*. *lalicollaris* appeared condensed, and the aperture was closed off by reticulopodia. These visual differences may be species specific or result from different functional conditions (i.e., moving state, adherence, and making new chambers). The reticulopodia at the aperture are not closed off in *A*. *kudakajimensis*, which may allow seawater to enter into the cytoplasm of *A*. *kudakajimensis* (Fig 2H). This organizational “pore” structure may also facilitate seawater influx and oxygen uptake at the shell surface [47].

### Cytoplasm in the test

Superimposed images from transmitted illumination and confocal microscopy showed the distribution of calcein-labeled cytoplasm and dinoflagellates inside the test (Fig 3A). The red autofluorescence from dinoflagellates was also observed in *A*. *kudakajimensis*. The dinoflagellates began to appear around the boundary between the outer and intermediate chambers. This observation is similar to the findings with *Sorites marginalis* [36].

Morphological differences in the cytoplasm at the boundary between outer and intermediate chambers were observed in *A*. *kudakajimensis*. Specifically, the shape of the cytoplasm changed from a branched structure in the outer chambers to a condensed structure in the intermediate chambers (Fig 3B). Similar cytoplasmic structures (i.e., vacuoles) and the seawater uptake in *Amphistegina lobifera* and *Ammonia tepida* have been previously observed using fluorescent indicators (i.e., fluorescein isothiocyanate; FITC-Dextran) [21, 24]. The reticulopodial distribution and vacuoles during chamber formation in living *A*. *tepida* were also visualized using FM 1–43 as a fluorescent membrane probe [24]. Using calcein AM, we showed that the cytoplasm in the outer chambers formed a large vacuolar structure (Fig 3C; dotted white encircled area approximately 20–50 μm in diameter), whereas the cytoplasm in the inner pert of chambers was condensed and contained small vacuoles (Fig 3C; white encircled area approximately 10–20 μm in diameter). The cytoplasm in the outer chambers was streaming and the vacuoles appeared to be in contractile motion (S3 Movie), whereas movement of the cytoplasm in the inner chamber was condensed and its movement was slow (Fig 3C and S3 Movie). These active cellular motions in the outer chambers may play a significant role in seawater transport from the outside of the test.

The condensed cytoplasm in the intermediate chambers to the inner chambers contains numerous dinoflagellates (Fig 3D and 3E). However, the embryonic apparatus was barely visible in this species because the discoid test is slightly concave toward the center. Therefore, this structure is located relatively deep (approximately 100 μm from the surface) and, at this depth, the chamber walls are thicker than those of the following cyclic chambers (Fig 3F). The cytoplasm in the embryonic apparatus was condensed in a seemingly similar manner to that which occurs from within the intermediate chambers to the inner chambers. The cytoplasm from the intermediate chambers to the embryonic apparatus may hold a large number of nuclei and organelles when compared with the other chambers [36].

## Conclusions

The cytoplasm and reticulopodia of only living *A*. *kudakajimensis* was visualized with calcein AM in a noninvasive manner. We showed that at least two types of reticulopodia exist in *A*. *kudakajimensis*: the straight bundle of reticulopodia that spreads from the aperture and the fine reticulopodia along the surface of the aperture and chamber walls. The fine reticulopodia formed a radially spreading, ring-shaped structure at the edge of the aperture. We also visualized the morphological/structural changes from branched cytoplasm in the outer chambers to a condensed one in the intermediate chambers. The cytoplasm in the outer chambers was continuously streaming, and the vacuoles showed contractile motion, while the cytoplasm in the intermediate chambers was condensed and moved slowly.

## Supporting Information

S1 Appendix Optimization of dye concentration and incubation time. (DOCX)

S1 Fig Confocal images and changes in the fluorescence intensity of calcein AM-labeled intrashell cytoplasm. **(A–C)** A time series of confocal images of the cytoplasm (in green) were superimposed on the corresponding bright-field images. The numbers to the left of the images indicate the dye concentration, and the numbers above the images indicate the recording time. The scale bars shown in (A–C) = 200 μm. Four regions of interest (ROIs 1–4) were defined in each image to analyze the concentration of dye in the cytoplasm. The yellow arrows in B point to the cytoplasm in some of the chamberlets was relatively easy to stain. The white arrow in C points the cytoplasm at the approximately 1–3 chambers from the outermost periphery. The red arrows indicate the cytoplasm of inner regions. **(D–F)** Changes in the mean fluorescence intensity (AU) within each ROI over time for initial dye concentrations of **(D)** 2 μM, **(E)** 10 μM, and **(F)** 50 μM. **(G, H)** Superimposed confocal and bright-field images of the cytoplasm at 1 h **(G)** and 24 h **(H)** after the addition of calcein AM (10 μM). Scale bars = 200 μm. **(I, J)** High-magnification images of the cytoplasm (green) and dinoflagellates (red) in the intermediate chamberlets after dye incubation for 1 h **(I)** and 24 h **(J)**. Scale bars = 50 μm.
(EPS)

S1 Movie Streaming reticulopodia of *A*. *kudakajimensis* in motion. (AVI)

S2 Movie 3D rotation of the aperture area. (AVI)

S3 Movie Fine reticulopodia on the surface of the test of *A*. *kudakajimensis*. (AVI)

S4 Movie Cytosolic streaming at the boundary from the outer to intermediate chambers of *A*. *kudakajimensis*. (AVI)
