# Correction: Cytological Observations of the Large Symbiotic Foraminifer *Amphisorus kudakajimensis* Using Calcein Acetoxymethyl Ester

**DOI:** 10.1371/journal.pone.0176803

**Published:** 2017-04-25

**Authors:** The PLOS ONE Staff

The last author's name is spelled incorrectly. The correct name is: Takashi Nakamura. The correct citation is: Ohno Y, Fujita K, Toyofuku T, Nakamura T (2016) Cytological Observations of the Large Symbiotic Foraminifer *Amphisorus kudakajimensis* Using Calcein Acetoxymethyl Ester. PLoS ONE 11(11): e0165844. https://doi.org/10.1371/journal.pone.0165844

The following information is missing from the Funding section: This work was supported by JST/JICA SATREPS, URL: http://www.jst.go.jp/global/english/about.html. The publisher apologizes for the errors.
