# Supplementary material for: Cytological Observations of the Large Symbiotic Foraminifer Amphisorus kudakajimensis Using Calcein Acetoxymethyl Ester
Source: PLoS One. 2016 Nov 3;11(11):e0165844. doi: 10.1371/journal.pone.0165844 (PMC5094710; doi:10.1371/journal.pone.0165844)
Supplement: S1 Appendix — (DOCX) [file pone.0165844.s001.docx]

**S1 Appendix. Optimization of dye concentration and incubation time**

We determined the optimal calcein AM concentration to use for our study in *A. kudakajimensis*. Time-lapse imaging was performed with different concentrations of calcein AM [i.e., 2 µM (n = 5), 10 µM (n = 6), and 50 µM (n = 5)] to assess the staining pattern of calcein AM. Four regions of interest (ROIs) were defined to determine the change in fluorescence over time. Each specimen was placed on a glass-based dish (No. 1S, thickness = 0.15–0.18 mm; Iwaki Glass, Tokyo, Japan). Time-lapse imaging was performed at a constant interval of 30 s immediately after filling the dish with 2 mL of the diluted calcein AM solutions. The solutions were prepared with FSW, and the experiment was completed at room temperature (approximately 25°C).

There were no green fluorescent signals from the cytoplasm of *A. kudakajimensis* at the start of the recording (S1 Fig. A–F, 0 min). Specimens incubated with 2 µM calcein AM showed minor diffuse fluorescent staining in the cytoplasm of the outer chambers during 120 min (S1 Fig. A and D, ROI 2). The cytoplasm in some chamberlets was weakly stained (S1 Fig. A, yellow arrwos). Specimens incubated with 10 µM showed calcein accumulation in the outer chambers after 30 min. The cytoplasm in some chamberlets was selectively stained in *A. kudakajimensis* (S1 Fig. B, yellow arrows). Calcein fluorescence gradually spread from the outside to the inside of the test during the first 30–60 min of incubation, and almost all of the chambers were stained with calcein AM after 120 min (S1 Fig. B).

The time course images with 50 µM calcein AM showed that chamberlets of approximately 1–3 chambers from the outermost periphery had strong fluorescent signal (S1 Fig. C, white arrow and E, ROI 2) after 30 min. Unlike the staining pattern observed at lower concentrations (i.e., 2 and 10 µM), this signal also began to be detected after 60 min in the cytoplasm of the inner regions of the test (S1 Fig. C, red arrow). After incubation for 120 min, calcein accumulated in the inner part of the test; however, the outer chambers were not well stained (S1 Fig. C and F, ROI 2).This unique staining pattern may be the result of cytoplasm shrinkage due to the cytotoxicity of higher calcein AM concentrations.

The background fluorescence (i.e., calcein AM containing filtered seawater) also increased continuously during 120-min incubation with 10 µM calcein AM (S1 Fig. B and E, ROI 1) and 50 µM calcein AM (S1 Fig. C and F, ROI 1). From these results we concluded that 10 µM calcein AM with a short incubation time (30–60 min) is appropriate to visualize the cytoplasm and reticulopodia in living *A. kudakajimensis* in a noninvasive manner.

We estimated the retention time of calcein in the cytoplasm of *A. kudakajimensis*. Specimens were incubated with 10 µM calcein AM for 30 min, and then the dye was removed. These specimens showed a bright and widespread calcein signal in the cytoplasm 1 h after the removal of calcein AM from the sea water (n = 3) (S1 Fig. G). After 24 h, the specimens showed a decreased florescent signal and an uneven calcein distribution pattern (S1 Fig. H). High magnification images taken 1 h after the incubation showed that the chamberlets were filled with cytoplasm (green), dinoflagellates (red), and vacuoles (dark area) (S1 Fig. I); however, the calcein was localized into granules after 24 h (S1 Fig. J). Thus, calcein AM staining method is not suitable for long-term observations of the cytoplasm of foraminifera.
